# Dynamic Interplay Between Autophagy and Oxidative Stress in Stem Cells: Implications for Regenerative Medicine

**DOI:** 10.3390/antiox14060691

**Published:** 2025-06-06

**Authors:** Daniela Rossin, Maria-Giulia Perrelli, Marco Lo Iacono, Raffaella Rastaldo, Claudia Giachino

**Affiliations:** Department of Clinical and Biological Sciences, University of Turin, 10043 Orbassano, Italy; mariagiulia.perrelli@unito.it (M.-G.P.); marco.loiacono@unito.it (M.L.I.); raffaella.rastaldo@unito.it (R.R.); claudia.giachino@unito.it (C.G.)

**Keywords:** ageing, antioxidant, autophagy, mitophagy, regenerative medicine, ROS, stem cell

## Abstract

The crosstalk between autophagy and oxidative stress is a cornerstone of stem cell biology. These processes are tightly interwoven, forming a regulatory network that impacts stem cell survival, self-renewal, and differentiation. Autophagy, a cellular recycling mechanism, ensures the removal of damaged organelles and proteins, thereby maintaining cellular integrity and metabolic balance. Oxidative stress, driven by the accumulation of reactive oxygen species (ROS), can act as both a signalling molecule and a source of cellular damage, depending on its levels and context. The interplay between autophagy and oxidative stress shapes stem cell fate by either promoting survival under stress conditions or triggering senescence and apoptosis when dysregulated. Recent evidence underscores the bidirectional relationship between these processes, where autophagy mitigates oxidative damage by degrading ROS-generating organelles, and oxidative stress can induce autophagy as a protective response. This crosstalk is critical not only for preserving stem cell function but also for addressing age-related decline and enhancing regenerative potential. Understanding the molecular mechanisms that govern this interplay offers novel insights into stem cell biology and therapeutic strategies. This review delves into the intricate molecular dynamics of autophagy and oxidative stress in stem cells, emphasizing their synergistic roles in health, disease, and regenerative medicine applications.

## 1. Introduction

Stem cells are essential for maintaining tissue turnover and regeneration due to their ability to self-renew and differentiate in response to physiological stimuli or injury. A key aspect of adult stem cell regulation is quiescence, a reversible non-proliferative state that preserves genomic integrity and long-term replicative potential. This state is maintained by both intrinsic and extrinsic signals from the surrounding niche, acting as a protective mechanism against replicative and metabolic stress [1]. Regeneration refers to the ability of stem cells to exit quiescence, proliferate, and differentiate to replace damaged or lost cells, thereby sustaining tissue turnover and enabling effective responses to injury [2].

Ageing disrupts the regulatory circuits that maintain quiescence, leading to either aberrant exit from the non-proliferative state or a deep, unresponsive quiescence. These alterations result in reduced activation capacity, increased vulnerability to oxidative stress, and a progressive decline in regenerative potential [3,4,5,6]. Ageing further impairs the stem cell microenvironment and promotes molecular damage [6]. 

Within this delicate balance, two fundamental cellular processes, autophagy and oxidative stress, play a central role in determining stem cell vitality. Autophagy is a conserved mechanism responsible for removing damaged mitochondria, aggregated proteins, and dysfunctional organelles, thereby maintaining cellular homeostasis and preventing senescence [7]. In contrast, the accumulation of reactive oxygen species (ROS) leads to cellular damage, senescence, and programmed cell death [8,9]. These two processes are tightly interconnected: autophagy mitigates oxidative stress through mitophagy, while excessive ROS levels can, in turn, activate autophagy as an adaptive response [10,11].

Multiple studies underscore the significance of autophagy and oxidative stress in governing stem cell fate decisions, including self-renewal, differentiation, and senescence. For instance, autophagy has been shown to sustain the metabolic requirements of hematopoietic stem cells (HSCs) by eliminating damaged mitochondria and preventing the accumulation of ROS [12,13,14,15,16,17,18]. Subsequently, in mesenchymal stem cells (MSCs), oxidative stress has also been shown to induce autophagy, thereby supporting mitochondrial integrity as a cellular survival strategy [19,20,21]. Moderate levels of ROS have been implicated as signalling molecules that drive stem cell differentiation, while excessive ROS levels can lead to apoptosis or senescence [22,23].

Maintaining a functional balance between these mechanisms is crucial to preserving regenerative capacity and preventing ageing-related dysfunctions, including neurodegenerative and cardiovascular disorders [24,25,26]. Therefore, targeting the interplay between autophagy and oxidative stress represents a promising strategy to enhance regenerative therapies.

This review explores the molecular mechanisms underlying the crosstalk between autophagy and oxidative stress in stem cells, with a particular focus on their implications for quiescence, ageing, and tissue regeneration in the context of regenerative medicine.

## 2. Autophagy in Stem Cells

Autophagy is essential for maintaining stem cells by regulating mitochondrial content to meet the cell’s metabolic demands. It prevents the build-up of damaged mitochondria and reduces ROS production. By protecting cells from metabolic stress, autophagy helps maintain genome stability and prevents stem cell death and senescence. Additionally, throughout its effect on epigenetic and metabolic programmes, autophagy exerts a role in determining cell fate, as well as regulating self-renewal, stem cell quiescence, activation, and differentiation. Dysregulated autophagy has been implicated in stem cell ageing, reduced regenerative capacity, and various diseases [27].

If not otherwise specified, the term autophagy in this review refers to macroautophagy, the most widespread and broadly characterized form of autophagy.

### 2.1. Molecular Pathways of Autophagy

The molecular pathways governing autophagy are complex and highly regulated. The autophagy programme is negatively regulated by the mechanistic target of rapamycin (mTOR) and positively regulated by AMP-activated protein kinase (AMPK). Autophagy begins with the formation of the phagophore, a double-membrane structure that engulfs cytoplasmic material for degradation. This process is regulated by a series of conserved autophagy-related (ATG) proteins and pathways that respond to cellular stress and metabolic cues [28].

#### 2.1.1. Initiation and Regulation

**Figure 1:**
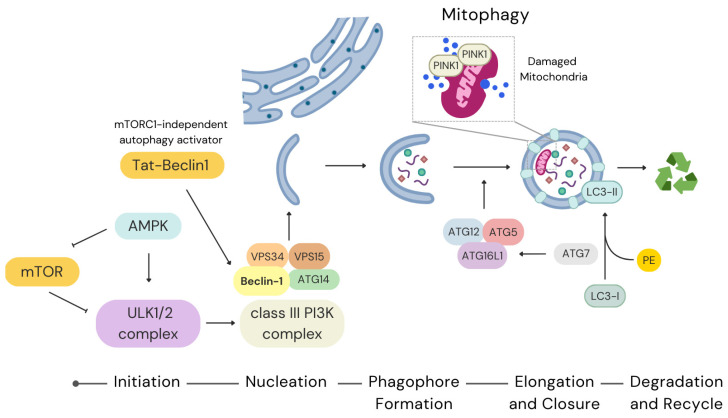
The phases of autophagy in stem cells. AMPK initiates the autophagic process by activation of the ULK1 complex and removing the inhibitory effect of mTOR. During nucleation, the activated ULK1/2 complex orchestrates the recruitment of the class III PI3K complex, which includes VPS34, VPS15, Beclin-1, and ATG14, with Tat-Beclin1 functioning as an mTORC1-independent autophagy inducer. The phagophore expands into the isolation membrane, which elongates and closes to form the autophagosome. This process is driven by two ubiquitin-like conjugation systems: one involving the ATG12–ATG5–ATG16L1 complex, and the other mediating the lipidation of LC3-I with phosphatidylethanolamine (PE) to generate LC3-II, a hallmark of autophagosome maturation. Both conjugation pathways critically depend on the E1-like enzyme ATG7.

The initiation of autophagy involves the formation of the Unc-51-like kinase 1/2 (ULK1/2) complex and Phosphatidylinositol 3-kinase (PI3K) protein complexes (Figure 1). Under nutrient-rich conditions, mTOR directly inhibits autophagy by phosphorylating ULK1, thereby preventing AMPK activation. Conversely, during energy stress, AMPK activates autophagy by phosphorylating ULK1 on a different serine residue. At the same time, AMPK can directly phosphorylate a subunit of the mTOR complex, thus inhibiting it [28,29,30]. In stem cells, mTOR signalling plays a pivotal role in balancing self-renewal and differentiation, making its regulation critical for maintaining stem cell homeostasis [14,28,31,32,33]. However, the effects of mTOR signalling vary depending on the type of stem cells involved [34,35].

#### 2.1.2. Nucleation and Phagophore Formation

Following activation, the ULK1 complex recruits the class III PI3K complex, including VPS34, VPS15, Beclin-1 (Becn1), and ATG14 to initiate phagophore nucleation. The generation of phosphatidylinositol-3-phosphate (PIP3), mainly by VPS34 at the nucleation site, serves as a platform for recruiting additional ATG proteins, facilitating the assembly of the autophagosome membrane (Figure 1) [28,36]. The role of Becn1 in stem cell autophagy is particularly important, as its dysregulation has been linked to impaired self-renewal and differentiation capacity in neural stem cells (NSCs) [37]. The role of the VPS34 complex has also been pointed out in endothelial precursor cells (EPCs). Specifically, the role of autophagy in cardiac protection under conditions of oxygen and glucose deprivation was studied in EPCs, which were recruited from bone marrow in response to cardiac ischemic events. The authors highlighted that the slight activation of autophagy through rapamycin-mediated inhibition of mTOR did not increase the survival of bone marrow-derived EPCs against injury. Conversely, the VPS34 complex activator Tat-Beclin1 (mTORC1-independent autophagy activator) restored the autophagy process with a consequent decrease in apoptotic EPCs [38].

#### 2.1.3. Elongation and Closure

The elongation and closure of the phagophore rely on two ubiquitin-like conjugation systems involving ATG12 and microtubule-associated protein 1A/1B-light chain 3 (LC3) (Figure 1). The ATG12-ATG5-ATG16L1 complex facilitates membrane expansion, while LC3 is conjugated to phosphatidylethanolamine (PE) to form LC3-II, which embeds into the autophagosome membrane [39]. LC3-II functions as a tag of autophagosome genesis and is crucial for cargo recognition and sequestration. In stem cells, LC3-mediated autophagy supports mitochondrial turnover, preventing ROS accumulation and preserving cellular function [40]. Recent findings underscored the importance of autophagy in the expansion and maturation of hematopoietic precursors [41].

ATG7 is an E1-like ligase, an essential protein in autophagosome biogenesis involved in Atg5-Atg12 and LC3-PE conjugation [42,43]. Using an in vitro model of embryonic stem cell (ESC) transition to epiblast-like cells (EpiLCs), it was recently demonstrated that dynamic changes in ATG7-dependent autophagy are essential for the naive-to-primed transition and germline specification [44].

Very recent experimental results confirmed that Atg7 is crucial for neurogenesis, though its influence can be exerted both on autophagy-dependent signalling pathways and through non-autophagic functions [45].

Stem cell viability is particularly dependent on the selective autophagic degradation of damaged mitochondria, known as mitophagy [46]. The PTEN-induced putative kinase 1 (PINK1)-Parkin pathway is a key regulator of mitophagy. Dysfunctional mitochondria show accumulated PINK1 on the outer mitochondrial membrane. This, in turn, recruits the E3 ubiquitin ligase Parkin to ubiquitinate mitochondrial proteins, marking the organelle for autophagic degradation [47]. Exploiting an innovative tool for investigating mitophagy, recent research demonstrated that PINK1 contributes to maintaining mitochondrial homeostasis and pluripotency in ESCs [48]. Stem cell mitophagy can also be mediated by two mitophagy receptor pathways, which involve BCL2-interacting protein 3 like/Nip3-like protein X (BNIP3L/NIX) and/or FUN14 domain containing 1 (FUNDC1) [49,50]. In mitophagy, damaged mitochondria, characterized by PINK1 accumulation, trigger their recognition and subsequent degradation. The final step involves autophagosome fusion with the lysosome, leading to the degradation and recycling of cellular components.

### 2.2. Transcriptional and Post-Transcriptional Regulation

Autophagy in stem cells is also regulated transcriptionally by factors such as Forkhead box O3 (FOXO3) and transcription factor EB (TFEB). Expression activation of Atg genes mediated by FOXO3 enhances the capability of stem cells to respond to stress and maintain homeostasis. Regulation of lysosomal biogenesis and autophagy mediated by TFEB functions through the activation of the transcription of genes involved in autophagosome formation and lysosomal function [51]. In neural stem and progenitor cells (NSPC), a network of Atg genes regulated by the transcription factor FOXO3 was identified while exploring the transcriptional programmes essential for cell function [52]. Further, it was found that FOXO3-mediated autophagy activation maintains redox balance during osteogenic differentiation [53] and protects HSCs from metabolic stress [54].

In a recent paper, the authors proposed a novel mechanism by which TFEB regulates the pluripotency of mouse embryonic stem cells (mESCs) through control of the pluripotency transcriptional network [55]. In another interesting paper, while trying to comprehend how quiescent long-term hematopoietic stem cells (LT-HSCs) perceive daily and stress-induced cues to transition into metabolically active progeny, the authors demonstrated that lysosomes, which function as advanced nutrient-sensing and signalling hubs, are modulated in a dual manner by TFEB and MYC to stabilize catabolic and anabolic activities essential for LT-HSC activation and lineage determination [56]. These findings establish TFEB-mediated lysosomal regulation as a key axis for orchestrating correct and synchronized stem cell fate decisions.

Post-transcriptional mechanisms, including non-coding RNAs such as microRNAs (miRNAs) and long non-coding RNAs (lncRNAs), further fine-tune autophagy.

For example, the expression of miR-145, miR-148a, and miR-185 was investigated in Wharton jelly multipotent stem cells (WJ-MSCs) from male and female donors in relation to the autophagic process and adipogenic/osteogenic differentiation potential. These miRNAs were chosen for their roles in regulating the stemness-related octamer-binding transcription factor 4 (OCT4) and DNMT1 gene expression and stem cell differentiation. Findings revealed a distinct regulatory mechanism involving the miR-148a/DNMT1/OCT4 autophagy pathway in male WJ-MSCs compared to female cells. However, no significant differences were observed in the expression of miR-145 and miR-185, which regulate differentiation [57]. Overall, these results highlight sex-based differences in WJ-MSC behaviour, offering insights into autophagy and stemness that could inform future clinical applications.

Further, lncRNAs can enhance autophagy by stabilizing the expression of key ATG proteins [58,59].

### 2.3. Autophagy and Stem Cell Ageing

Recent studies establish a strong connection between autophagy and stem cell ageing. Among others, the role of chaperone-mediated autophagy in ageing biology and stem cell rejuvenation has been reviewed in [60,61]. Ageing is associated with a decline in autophagic activity, causing the accumulation of damaged organelles and macromolecules. This contributes to stem cell exhaustion and a reduction in regenerative capacity. Enhancing autophagy through genetic or pharmacological means was shown to rejuvenate aged stem cells and improve their function.

The mechanisms underlying the decline of autophagy with age remain unclear and are the focus of ongoing research. Recent studies on aged muscle stem cells (MuSCs) and HSCs have shown that autophagy deteriorates with ageing, leading to impaired stem cell activity [13,62]. Both MuSCs and HSCs lose their regenerative potential with age, and old stem cells exhibit autophagy deficiencies, characterized by the autophagic vesicle enhancement, elevated intracellular p62 levels, increased LC3II expression, and the presence of ubiquitin-positive inclusions. However, treatment with rapamycin and spermidine restores autophagic function, mitigating autophagy-related deficiencies in both systems [13,62].

A previous study also highlighted that autophagy, particularly Atg7, is essential for meeting the bioenergetic demands required for the activation of quiescent MuSCs following injury, a process mediated by SIRT1 [63]. These findings confirm that autophagy is critical for preserving stemness in both MuSCs and HSCs, though the mechanisms appear to vary depending on the cellular niche.

In MSCs, instead, a dual role of autophagy has emerged in the context of ageing [64]. Under normal conditions, p62-dependent autophagy selectively degrades GATA4, a key regulator of the senescence-associated secretory phenotype (SASP), thereby suppressing senescence. However, when senescence-inducing stimuli arise, the interaction between GATA4 and p62 gradually declines, leading to the accumulation of GATA4, which subsequently activates the SASP transcriptionally [64].

### 2.4. Autophagy in Disease and Therapy

Dysregulated autophagy has been implicated in several stem cell-related diseases, like neurodegenerative diseases and cancers [65]. For instance, the expression of Presenilin 1 (PS1) progressively declines in the aged human brain, and PS1 mutations are the leading cause of early-onset familial Alzheimer’s disease. While PS1-knockout and PS1 mutant neurons exhibit dominant autophagy-related phenotypes, it remains unclear whether autophagy in NSCs is significantly impaired by PS1 deficiency. Recently, authors utilized CRISPR/Cas9-based gene editing to produce human induced pluripotent stem cells (iPSCs) that were PS1-knockout in either heterozygosis or homozygosis, after which these cells differentiated into NSCs. In PS1-deficient NSCs, autophagosome formation as well as expression of mRNAs and proteins related to the autophagy–lysosome pathway (ALP), were reduced [66]. Mechanistically, inhibition of ERK/CREB signalling and activation of GSK3β played critical roles in suppressing TFEB expression in PS1-knockout NSCs [66]. These results indicate that PS1 deficiency leads to autophagy repression in human NSCs by downregulating ERK/CREB signalling, thus contributing to a major understanding of the role of PS1 in autophagy regulation.

Targeting autophagy pathways offers a promising therapeutic strategy for enhancing stem cell survival and function. Pharmacological activators of autophagy, such as rapamycin, have been explored for their ability to improve stem cell transplantation outcomes and tissue regeneration [36,67]. Transplantation of satellite cells, along with lineage-tracing approaches, revealed that hypercapnia disrupted satellite cell replication, activation, and myogenic potential. Sequencing analyses, performed both in bulk and at the single-cell level, revealed that hypercapnia altered autophagy, senescence, and other key cellular pathways [67]. In hypercapnic mice, rapamycin restored satellite cell autophagy flux, enhanced their activation and replication rates, and improved myogenic capability post-transplantation. These findings demonstrate that in hypercapnic mice treated with rapamycin, the functions of satellite cells were restored through AMPK activation and mTOR inhibition [67].

In another paper, authors faced the problem of stem cell transplantation after infarction, which is often impaired by the poor survival and engraftment of cells within the harsh microenvironment of the damaged heart. The study examined whether rapamycin-induced autophagy activation could enhance the survival of transplanted MSCs [68]. Rat bone marrow MSCs preconditioned with rapamycin exhibited reduced apoptosis and enhanced secretion of key factors, including hepatocyte growth factor (HGF), insulin-like growth factor-1 (IGF-1), stem cell factor (SCF), stromal cell-derived factor-1 (SDF-1), and vascular endothelial growth factor (VEGF). Moreover, rapamycin treatment significantly increased autophagic activity and lysosome production in MSCs. Following transplantation into a rat ischemia/reperfusion model, a few transplanted MSCs were observed to differentiate into cardiomyocytes (at least apparently) and endothelial cells in the damaged hearts [68]. These findings indicate that a low level of autophagy activation through rapamycin preconditioning sustains the engraftment and differentiation of transplanted cells, ultimately promoting myocardial repair and improving cardiac function.

Intriguingly, the regulation of autophagy can impact the regenerative potential of MSCs, both affecting MSC properties and through MSCs’ capacity to modulate autophagy of cells in damaged tissues/organs [69]. For example, MSCs can modulate autophagy in immune cells related to injury-induced inflammation, diminishing their survival, proliferation, and function. As a consequence, this favours the clearance of inflammation. Furthermore, MSCs can modulate autophagy in endogenous progenitor cells, fostering their proliferation and differentiation. This contributes to the repair of altered tissue [69]. A greater clarification of the pathways through which MSCs regulate the autophagy of various types of target cells and how autophagy can impact MSCs’ clinical potentialities will contribute to a broader perspective for the therapeutic application of MSCs in multiple pathologies.

In summary, dysregulated autophagy is a crucial element in the pathogenesis of various stem cell-related diseases. Modulating autophagy pathways presents a promising path for therapeutic treatments aimed at enhancing stem cell survival, improving transplantation outcomes, and promoting tissue regeneration.

## 3. Oxidative Stress in Stem Cells

Oxidative stress, characterized by an imbalance between ROS production and antioxidant defences, significantly impacts stem cell function. While moderate ROS levels serve as signalling molecules promoting stem cell differentiation, excessive ROS can lead to detrimental effects, including DNA damage, protein oxidation, and lipid peroxidation, ultimately impairing stem cell function [70].

ROS are chemically reactive molecules derived from oxygen, including superoxide anions (O_2_^•−^), hydrogen peroxide (H_2_O_2_), and hydroxyl radicals (^•^OH). Electrons escaping from the electron transport chain react with molecular oxygen to form O_2_^•−^, which are then converted to less reactive species via enzymatic and non-enzymatic antioxidant defences [71].

### 3.1. Sources and Regulation of ROS

The primary source of ROS in all cells, including stem cells, is the mitochondria, particularly during oxidative phosphorylation (OxPhos) [72,73]. About 0.1–0.2% of mitochondrial oxygen consumption produces ROS as byproducts of the respiratory chain [74]. The exact amount of ROS produced during mitochondrial respiration varies significantly based on cell type, environmental conditions, and mitochondrial activity [75]. Consequently, cells regulate ROS levels by modulating mitochondrial function and metabolic pathways. Precisely, ROS production can be reduced by drawing substrates away from OxPhos, as well as by boosting pathways that restore oxidized glutathione (GSH), like the pentose phosphate pathway.

ROS generated by OxPhos, and especially H_2_O_2_, are key regulators of stem and progenitor cell function in both physiological and pathological contexts. In quiescent HSCs, low H_2_O_2_ levels help maintain stemness, whereas increased H_2_O_2_ levels within HSCs or their niche promote survival, proliferative activity, differentiation, and cell migration [73]. Conversely, in pathological conditions such as ageing, atherosclerosis, heart failure, hypertension, and diabetes, excessive ROS levels contribute to an inflammatory and oxidative environment, triggering damage and apoptosis in stem and progenitor cells [73].

Another key ROS source in stem cells is the nicotinamide adenine dinucleotide phosphate oxidase (NOX) family, which contributes to redox balance together with the antioxidant systems [76,77]. Unlike mitochondrial ROS, NOXs intentionally produce ROS by transferring electrons from the nicotinamide adenine dinucleotide phosphate (NADPH) to O_2_, thus generating superoxide (O_2_^•−^) which can be converted to hydrogen peroxide (H_2_O_2_) by superoxide dismutase (SOD). A distinct NOX subcellular localization results in compartmentalized ROS production, enabling precise redox signalling regulation [78]. This redox equilibrium is closely linked to the regulation of stem cell fate, influencing self-renewal, proliferation, and differentiation, which are critical processes in embryonic development, adult tissue regeneration, and cell therapy applications [77]. Although cytochrome P450, xanthine oxidase (XO), and nitric oxide synthases (NOS) are minor ROS sources, they can influence redox balance [77].

### 3.2. Antioxidant Defence Mechanisms

Stem cells hold intrinsic antioxidant defence mechanisms which include both enzymatic systems such as SOD, catalase (CAT), and glutathione peroxidase (GPx), and non-enzymatic molecules like GSH, vitamins C and E, and flavonoids. These systems work synergistically to neutralize ROS and maintain redox balance [26,79,80,81].

HSCs are highly sensitive to intracellular redox balance, requiring minimal ROS levels and NOX expression to maintain quiescence [82]. Research indicates that quiescent, proliferative, and differentiated stem cells exhibit varying ROS levels due to metabolic differences. Maintaining low ROS levels, regulated by both internal and external factors, is crucial for stem cell self-renewal, migration, development, and cell cycle regulation [83,84,85]. However, elevated ROS levels drive HSCs out of quiescence, promoting differentiation at the expense of self-renewal. If left unchecked, this imbalance can deplete the HSC pool and contribute to disease development [86].

Conversely, MSCs have been shown to exhibit resistance to oxidative and nitrosative stress, at least in vitro, a capability linked to their constitutive expression of antioxidant enzymes like SOD1, SOD2, CAT, and GPx, as well as abundant levels of the antioxidant GSH [87]. Depleting GSH compromises their tolerance to oxidative stress. Additionally, MSCs constitutively express heat shock protein 70 (HSP70) and sirtuins (SIRT), especially SIRT1, 3, and 6 [88], which may further contribute to their resilience against oxidative and nitrosative damage. SIRT1 promotes MSC survival under H_2_O_2_-induced oxidative stress [89]. Similarly, SIRT6 has been implicated in reducing oxidative damage and basal ROS levels in MSCs by promoting the production of antioxidants, such as heme oxygenase-1 (HO-1) [90]. Overexpressing HO-1 has been shown to mitigate ROS accumulation and cellular senescence in SIRT6-deficient MSCs, highlighting its critical role in MSC survival under oxidative conditions [90].

Beyond their inherent antioxidant defences, MSCs can also adapt to redox stress. When treated with lipopolysaccharides (LPSs), MSCs produce oxidative and nitrosative free radicals, further demonstrating their dynamic response to environmental stressors [88].

### 3.3. Impacts of Oxidative Stress on Stem Cell Function

**Figure 2:**
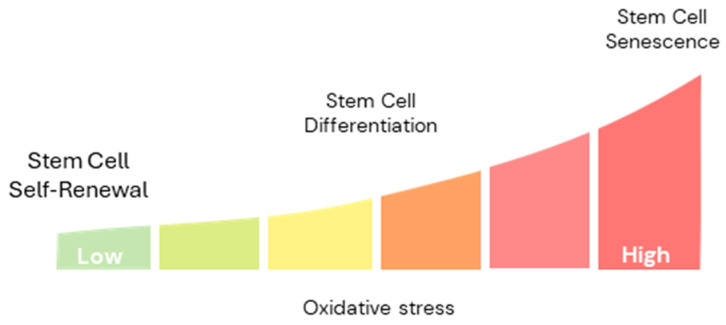
Oxidative stress gradient regulates stem cell fate. Low levels of oxidative stress promote stem cell self-renewal, while moderate levels drive differentiation. However, excessive oxidative stress leads to cellular damage and senescence, impairing stem cell function and regenerative potential. Maintaining an optimal oxidative balance is crucial for sustaining stem cell homeostasis and function.

In stem cells, the equilibrium between self-renewal and differentiation is influenced by the redox balance regulation in coordination with metabolism [22]. Studies indicate that ROS amounts remain limited in niches where stem cells undergo self-renewal, while they increase as stem cells differentiate (Figure 2) [91,92]. In various stem cell types, both excessive and insufficient ROS levels can impair regenerative potential through the reduction in proliferation activity, differentiation potential, and self-renewal maintenance [90,93,94,95,96]. In particular, quiescent adult stem cells in their niches display ROS signalling suppression caused by antioxidant expression induced by high transcription factor levels, like nuclear factor erythroid 2–related factor 2 (Nrf2), while proliferating cells show activated growth factor kinase signalling and altered redox states consisting of ROS signalling activation caused by antioxidant reduction [97]. Therefore, maintaining ideal ROS levels is essential for appropriate stem cell function. However, primary neural progenitors with proliferative, self-renewing, and multipotency characteristics similar to NSCs unexpectedly maintained high ROS levels and exhibited strong responsiveness to ROS stimulation [93]. A ROS-driven increase in self-renewal and neurogenic capability was found to rely on the PI3K/AKT axis. Indeed, reduced intracellular ROS amounts in response to pharmacological and genetic interventions disrupted NSC and progenitor activity in vitro and in vivo [93]. AKT double-deficient HSCs display reduced ROS and remain quiescent, and restoring ROS pharmacologically rescues their differentiation capacity [94]. These results highlight AKT1 and AKT2 as essential regulators of HSC function and suggest that disrupted ROS homeostasis may contribute to impaired hematopoiesis. Notably, a modest rise in basal ROS levels has been shown to promote MSC proliferative and migratory activities through the triggering of ERK 1/2- and JNK-1/2-mediated signalling pathways [98,99,100].

#### 3.3.1. ROS and Stem Cell Differentiation

Moderate levels of ROS act as secondary messengers in cellular signalling pathways, promoting stem cell differentiation. For example, ROS-mediated activation of the p38 MAPK (p38) and NF-κB pathways has driven MSC differentiation into osteoblasts [101]. The pluripotency markers of ESCs, including OCT4, NANOG, and sex-determining region Y-box 2 (SOX2), are downregulated in the presence of elevated ROS levels, promoting ESC differentiation toward mesodermal and endodermal lineages. Remarkably, this pluripotency can be restored through antioxidant treatment. These effects are regulated by several members of the mitogen-activated protein kinase (MAPK) family, which modulate numerous signalling pathways [102]. Moliner et al. demonstrated that the augmented differentiation of ESCs into neurons within spheres correlates with enhanced expression of genes linked to mitochondrial metabolism and ROS production [103]. In cortical clones, ROS are promptly generated in the culture condition, driving differentiation into both large pyramidal-like neurons and calretinin-expressing neurons [104]. Similarly, Le Belle et al. found that pharmacological inhibition of the NOS enzyme led to increased oxidative stress, which, in turn, stimulated neuroepithelial stem (NES) cell activity and self-renewal [93]. Moreover, ROS-driven neurogenesis relies on the activation of JNK signalling [105]. Spitkovsky et al. observed an increase in mitochondrial biogenesis during in vitro mESC cardiogenic differentiation. They also showed that complex III activity drives essential Ca^2^⁺ oscillations to initiate this differentiation, independent of ATP production [106].

Conversely, Chung and colleagues evidenced how the shift from anaerobic glycolysis to aerobic OxPhos was essential for proper mESC differentiation toward a functional cardiac phenotype [107,108].

#### 3.3.2. Excessive ROS and Stem Cell Damage

On the other hand, excessive ROS disrupts this balance, leading to DNA damage, protein oxidation, and lipid peroxidation, ultimately impairing stem cell function and leading to stem cell senescence [109]. Jang and Sharkis demonstrated that mouse bone marrow-derived HSCs could be categorized into ROS^high^ and ROS^low^ cell groups based on the intensity of dichlorodihydrofluorescein (DCF) staining [82]. ROS^high^ HSCs showed a diminished capacity to generate long-term cells in vitro, as well as a reduced ability to sustain long-term engraftment upon transplantation compared to ROS^low^ HSCs. In NSCs, excessive ROS leads to oxidative DNA damage, triggering the activation of the p53 signalling pathway. This activation resulted in cell cycle arrest, senescence, or apoptosis, thereby impairing the regenerative capacity of NSCs, highlighting the sensitivity of these cells to oxidative stress [110]. However, minimal p53 signalling is key to these cells. In a recent paper, Navarro et al. investigated the p53 role in human brain development, using human iPSC-derived NSCs, along with brain organoids. They knocked down p53 in human iPSC-derived NES cells derived from iPSCs and observed that the knocked down cells suddenly developed centrosome amplification and genomic instability. Additionally, these cells exhibited diminished proliferative activity, downregulation of OxPhos-related genes, and increased glycolytic activity [111]. Furthermore, knocked-down NSCs differentiated into neurons at an accelerated rate, displaying characteristics of more mature neurons compared to controls. In brain organoid models of cortical neurogenesis, the loss of p53 led to a disorganized stem cell layer, a reduction in cortical progenitor cells and neurons, and a similar downregulation of OxPhos-related genes observed in NES cells [111]. Overall, these findings highlight a key role for p53 in ensuring genomic stability in NSCs, regulating neuronal differentiation, and maintaining proper structural organization and metabolic gene expression in neural progenitors within organoids.

Despite the significance of oxidative stress, its epigenetic effects on stem cells remain largely unexplored [112]. Epigenetic regulation appears to involve a complex interplay of multiple factors and coordinated signalling pathways. While an optimal stem cell model that accurately reflects epigenetic dynamics in the presence of ROS is still lacking, high-throughput sequencing could provide, in the future, valuable insights into the global epigenetic changes induced by ROS [112].

### 3.4. Therapeutic Implications

Understanding the dual role of ROS in stem cell biology is crucial for developing therapeutic approaches. To lower oxidative stress in stem cells, several strategies have been developed, including antioxidant therapies, metabolic reprogramming, activation of the Nrf2 pathway, and optimization of culture conditions.

Such approaches aim to maintain the delicate balance of ROS required for normal stem cell function and to prevent oxidative stress-induced impairments.

#### 3.4.1. Antioxidant Therapies

Administration of exogenous antioxidants such as N-acetylcysteine (NAC), ascorbic acid 2-phosphate (AAP), edaravone, and atorvastatin has shown promise in reducing oxidative damage and enhancing stem cell viability [113,114,115]. NAC, a thiol-containing antioxidant and GSH precursor, attenuates oxidative stress by scavenging free radicals. Through a systematic analysis, the authors uncovered the synergistic protective mechanism of NAC and AAP on human MSCs (hMSCs) exposed to H_2_O_2_-induced oxidative stress. The combined NAC and AAP (NAC/AAP) treatment mitigates ROS production, preserves mitochondrial membrane potential, and reduces oxidative stress-induced mitochondrial fission and fragmentation [116]. However, it is interesting to note that the effects of NAC are concentration-dependent. For example, a study explored the effects and mechanisms of NAC on human dental follicle stem cells (hDFSC) which possess MSC characteristics [114]. These authors evaluated MSC properties and their role in alveolar bone regeneration when exposed to different NAC doses. Treatment with 5 mM NAC enhanced hDFSC proliferation, reduced senescence, and increased the expression of stem cell- and immune-related markers, resulting in the strongest osteogenic differentiation. Different doses also helped maintain stem cell features. The authors found that optimizing NAC concentration augments hDFSC characteristics, particularly osteogenesis, through PI3K/AKT/ROS signalling [114]. In another paper, the authors demonstrated that only ROS^high,^ and not ROS^low^, HSCs became exhausted after the third transplantation [82]. Such impairments were linked to heightened activation of p38 and mTOR, which could be mitigated by treating the cells with an antioxidant, a p38 inhibitor, or rapamycin. Moreover, exposing mouse bone marrow-derived HSCs to high concentrations of buthionine sulfoximine, an inhibitor of GSH metabolism, significantly decreased HSC clonogenicity [94,117].

The treatment time windows of the antioxidant edaravone were evaluated in human umbilical cord mesenchymal stem cells (hUCMSCs) exposed to an LPS/H_2_O_2_ challenge. The therapeutic effects and underlying mechanisms of edaravone-treated hUCMSCs were then studied in vivo using a murine model of acute liver failure , where increased implanted stem cell numbers and improved hepatic function were evidenced [118].

Atorvastatin-treated MSCs derived from the bone marrow showed improved survival upon implant in a setting of renal ischemia–reperfusion injury, accompanied by injury amelioration outcomes [119]. Similarly, through activation of NOS, atorvastatin enhanced the efficacy of MSC treatment for swine myocardial infarction [120].

However, since different cell types showed different oxidative stress threshold susceptibility, further investigation is required to evaluate the dose-depentent effects of antioxidants and their cell type-specific actions before extensive clinical application.

#### 3.4.2. Metabolic Reprogramming

Shifting stem cell metabolism from OxPhos to glycolysis can reduce mitochondrial ROS production. As mouse PSCs transition from the naïve to the primed pluripotent state, they naturally decrease their reliance on OxPhos and shift to high glycolytic activity. This occurs due to the elevated expression of glucose transporters, resulting in increased glucose uptake and glycolysis [121]. Research using genetically modified mouse models and progress in metabolomic analysis, particularly in HSCs, have enhanced our understanding of how metabolic signals regulate stem cell self-renewal. Multiple stem cell types primarily depend on anaerobic glycolysis, while their function is also influenced by bioenergetic signalling, the AKT–mTOR pathway, glutamine metabolism, and fatty acid metabolism [122]. Quiescent adult stem cells typically exhibit a preference for glycolysis, even under aerobic conditions, to minimize oxidative stress. This metabolic reprogramming is crucial for maintaining stem cell function and has been observed during processes such as neuronal differentiation [123]. The mechanisms underlying metabolic reprogramming during neuronal differentiation have remained unclear for a long time, until authors found that the passage from aerobic glycolysis in neural progenitor cells (NPCs) to OxPhos in mature neurons is defined by the downregulation of hexokinase (HK2) and lactate dehydrogenase (LDHA) proteins, along with a shift in pyruvate kinase splicing from PKM2 to PKM1. This shift coincides with a sharp decline in the amount of c-MYC and N-MYC, working as transcriptional activators of HK2 and LDHA [123]. Forcing the continuous expression of HK2 and LDHA along cell maturation determines neuronal cell death, highlighting the necessity of shutting down aerobic glycolysis for neuronal survival. Additionally, the metabolic regulators PGC-1α and ERRγ show a significant upregulation during differentiation, supporting the sustained transcriptional activation of metabolic and mitochondrial genes. Notably, these gene expression levels remain unmodified compared to NPCs, suggesting specific transcriptional regulation in proliferative versus post-mitotic differentiation states [123]. Furthermore, mitochondrial mass expands in proportion to neuronal growth, pointing to an as-yet unidentified mechanism that links mitochondrial biogenesis to cell size [123]. Recent research indicates that small non-coding RNAs, such as piRNAs, are involved in regulating this metabolic shift, further highlighting the complexity of metabolic control in stem cells [124].

#### 3.4.3. Activation of Nrf2 Pathways

The Nrf2 pathway is a crucial regulator of cellular antioxidant responses. Pharmacological activation of Nrf2 upregulates the expression of antioxidant genes, protecting stem cells from oxidative stress and improving their regenerative capacity [125]. NAC has been shown to exert its protective effects, partially, through the activation of the Nrf2 pathway, leading to increased GSH synthesis and enhanced cellular defence mechanisms. However, to some extent, ROS production is necessary for various stem cell types to transition from quiescence to activation and differentiation. Therefore, Nrf2-mediated regulation of redox-related genes has to be precisely balanced to accommodate the functional needs of stem cells, depending on their dormant or active state. One example of Nrf’s role in redox balance involves peroxiredoxins 1 and 6 (PRDX1/6), which play a key role in reducing intracellular H_2_O_2_. In human embryonic stem cells (hESCs), the maintenance of stemness requires ROS suppression, as PRDX1 deletion leads to ROS accumulation and diminished stem cell properties [126]. In contrast, dental pulp stem cells (DPSCs) require a reduction in PRDX6 levels to undergo osteogenic differentiation, as PRDX6 overexpression inhibits dental bone development [127]. These findings highlight how different stem cell niches necessitate distinct ROS levels based on their functional context, with Nrf2 and its redox targets playing a central role in mediating ROS-dependent shifts in stem cell status.

#### 3.4.4. Improved Culture Conditions

Mimicking the hypoxic conditions of the stem cell niche by reducing oxygen levels in culture has long been shown to decrease ROS levels and to help maintain the stem cell undifferentiated state [128,129]. Further, culturing stem cells under low oxygen conditions enhances their therapeutic potential [130]. In the case of adipose-derived stem cells (ASCs), for instance, it was found that, compared to the hyperoxia group, cells in the physioxia group demonstrated enhanced proliferation, migration, and angiogenesis, along with reduced senescence and apoptosis [131]. The improved survival rate of ASCs cultured under physioxia was observed in both in vitro and in vivo ischemia models. Metabolic reprogramming analysis revealed a reduction in mitochondrial mass, an increase in intracellular pH alkalization, an enhanced glucose uptake, and glycogen synthesis [131]. These findings suggest that physioxia provides a more favourable environment for culturing ASCs for transplantation, as it preserves their native bioactivities while preventing hyperoxia-induced damage.

Pyruvate, a crucial byproduct of glycolysis, plays a crucial role in stem cell metabolism. Recently, hESC culture shows that higher exogenous pyruvate levels shift metabolism toward OxPhos and promote mesoderm and endoderm differentiation [132]. However, pyruvate production and its mitochondrial metabolism are crucial for mesoderm differentiation. TCA-cycle metabolites cannot replace their role [132]. Additional investigation revealed that pyruvate elevates the AMP/ATP ratio, activates AMPK, and regulates the mTOR pathway to promote mesoderm differentiation [132,133]. These results highlight that, besides its influence on hESC metabolism, exogenous pyruvate also modulates key signalling pathways in stem cell differentiation.

Beyond glucose metabolism, fatty acid metabolism is also essential for the terminal stages of hESC endodermal differentiation [134]. Increased fatty acid β-oxidation and decreased adipogenesis occur due to the AMPK-mediated phosphorylation of the adipogenic enzyme acetyl-CoA carboxylase [134]. As a result, inhibiting fatty acid synthesis through AMPK agonists exerts a crucial role in facilitating and regulating human endodermal differentiation. Recent research has identified the PI3K/AKT axis as a crucial metabolic signalling target for enhancing stem cell survival, offering new insights into optimizing long-term MSC culture [135]. Further, in bone marrow stem cells, the natural ginseng extract Rg1 activates the PI3K/AKT pathway, preventing MSC ageing and enhancing antioxidant capacity, thus making Rg1 a promising regenerative therapy [136].

In summary, oxidative stress exerts a complex role in stem cell biology, influencing both differentiation and survival. Maintaining ROS at optimal levels is essential for preserving stem cell function and developing effective therapeutic interventions.

## 4. Crosstalk Between Autophagy and Oxidative Stress

The interplay between autophagy and oxidative stress is a dynamic and tightly regulated process that significantly influences stem cell fate. Autophagy and oxidative stress are interdependent, with each modulating the other to maintain cellular homeostasis.

As reported, ROS-generating organelles such as mitochondria, as well as peroxisomes, organelles that undergo cellular pathways involved in lipid metabolism, can be degraded through autophagy. The process relies on the binding of ubiquitinated proteins to specific autophagy receptors (SQSTM1, NBR1, and NDP52). The autophagic process also withdraws unfolded proteins through specialized pathways like chaperone-mediated autophagy. Further, autophagy-dependent Nrf2 activation promotes expression of antioxidant genes to remove excess intracellular ROS.

On the other hand, a dual role of ROS is recognized in autophagy induction and inhibition. ROS trigger transcription factors like p53, HIF1A, and Nrf2 to induce the expression of Atg genes. In addition, ROS-mediated oxidation of Atg4 inhibits its LC3-PE deconjugation activity, resulting in the accumulation of autophagic LC3-II isoforms, which facilitate autophagosome formation. ROS directly oxidize cysteine residues on the α and β subunits of AMPK, triggering phosphorylation of the ULK1 complex and inhibition of PI3K-AKT-mTORC1 signalling pathway, both of which promote autophagy. In contrast, ROS can inactivate PTEN, which negatively affects the PI3K-AKT-mTORC1 pathway, ultimately suppressing autophagy. Moreover, the inhibition of autophagy is also mediated by oxidation of catalytic thiols on Atg3 and Atg7 by ROS, inhibiting LC3 lipidation [137,138].

In a feedback mechanism, autophagy helps reduce ROS levels and remove damaged organelles, including mitochondria, through mitophagy. Damaged mitochondria are selectively targeted for degradation by binding to LC3 and being enclosed within the autophagosome. The autophagosome then fuses with a lysosome, forming an autolysosome where the mitochondria are broken down and recycled [137].

In stem cells, this crosstalk is mediated through shared molecular pathways, including mitochondrial quality control, transcriptional regulators, and signalling molecules.

### 4.1. Mitochondrial Quality Control

Mitochondria are a predominant source of ROS within cells, and their dysfunction can exacerbate oxidative stress, leading to cellular damage and impaired stem cell function. Autophagy, particularly mitophagy, is crucial for maintaining mitochondrial quality by selectively removing damaged mitochondria that generate excessive ROS.

This quality control process is mediated by the PINK1-Parkin pathway, which tags defective mitochondria for degradation via ubiquitination. Additionally, other mediators such as BCL2 Interacting Protein 3 (BNIP3) and FUNDC1 regulate hypoxia-induced mitophagy, ensuring cellular adaptation to low oxygen environments [139]. Stem cells with impaired mitophagy exhibit elevated oxidative damage, resulting in diminished functionality and increased susceptibility to stress [49,140].

In HSCs and ESCs, a lower mitochondrial count is linked with decreased dependence on aerobic metabolism, leading to lower ROS production [121,141]. Additionally, mitophagy contributes to maintaining low ROS levels during somatic cell reprogramming into iPSCs via ATG proteins such as PINK1 and ATG3 [142,143,144]. Notably, the loss of PINK1-dependent mitophagy significantly reduces the speed and efficiency of iPSC reprogramming from mouse embryonic fibroblasts [143]. Consistent with this, iPSCs derived from Pink1-knockout mice exhibit decreased glycolytic metabolism and an increased tendency toward differentiation.

A recent study delved into the mechanisms of mitochondrial function maintenance in MuSCs [145]. In this research, the authors investigated mitophagy dynamics in MuSCs across different differentiation states of myogenesis and assessed the role of PINK1 in preserving their regenerative potential. Their findings reveal that quiescent MuSCs actively express mitophagy-related genes, show detectable mitophagy flux, and exhibit significant mitochondrial localization within autophagolysosomes, processes that suddenly decline upon activation [145]. In mice, genetic deletion of Pink1 disrupts Parkin recruitment to mitochondria and impairs mitophagy in quiescent MuSCs. This loss is associated with premature activation and commitment, leading to a decline in self-renewal and an ongoing loss of muscle regeneration, despite normal proliferation and differentiation capabilities. Additionally, authors found that the weakened fate decisions in Pink1-deficient MuSCs can be rescued by scavenging redundant mitochondrial ROS [145]. These results exemplify the critical contribution of mitophagy in MuSC regulation and establish PINK1 as a key mediator of mitochondrial integrity and stem cell fate.

Another recent research revealed that *O*-linked *N*-acetylglucosamine (O-GlcNAc) transferase (OGT) critically controls the stress response of HSCs by securing mitochondrial quality through PINK1-dependent mitophagy [146]. OGT is a unique enzyme that adds O-GlcNAc modifications to target proteins, playing a crucial role in regulating several cellular processes across different cell lineages. Nevertheless, its function in hematopoietic stem and progenitor cells (HSPCs) has remained unclear for a long time. The authors used Ogt conditional knockout mice to demonstrate that OGT is crucial for HSPC maintenance. They found that Ogt is particularly expressed in HSPCs, and its elimination causes a rapid loss of these cells, accompanied by increased ROS levels and apoptosis [146]. Notably, Ogt-deficient HSCs lose their quiescent state, fail to sustain themselves in vivo, and become highly susceptible to regeneration stress. Moreover, Ogt-deficient HSCs collect high amounts of faulty mitochondria because of impaired mitophagy, which is linked to reduced expression of the essential mitophagy regulator PINK1 via dysregulated H3K4me3 [146]. Importantly, excessive production of PINK1 re-establishes mitophagy and rescues the population of Ogt-deficient HSCs [146]. These findings underline the crucial role of OGT in maintaining HSC function and mitochondrial quality control.

Conversely, oxidative stress can also serve as a trigger for mitophagy, acting as a protective mechanism against mitochondrial dysfunction. Oxidative stress activates Nrf2, a transcription factor that upregulates the expression of Atg genes, including those required in mitophagy. This creates a feedback loop where ROS-induced mitophagy mitigates oxidative stress, thereby safeguarding stem cell viability and functionality. Moreover, the interdependence of autophagy and oxidative stress plays a pivotal role in maintaining stem cell homeostasis and preventing premature ageing [147].

Further research is needed, however, to uncover the molecular mechanisms underlying mitophagy-driven metabolic reprogramming and to explore its potential as a strategy for regulating stem cell quiescence and differentiation.

### 4.2. Transcriptional Regulation

**Figure 3:**
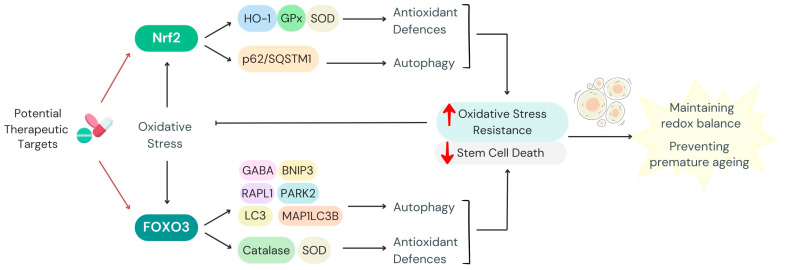
Redox balance triggered by FOXO3 and Nrf2 in stem cells. In response to oxidative stress, the transcription factors FOXO3 and Nrf2 are activated and orchestrate distinct yet complementary cryoprotective programmes. FOXO3 enhances the expression of different autophagy-related genes (e.g., LC3, BNIP3, MAP1LC3B, GABA, RAPL1, and PARK2) and antioxidant enzymes (e.g., catalase and SOD). Nrf2 upregulates antioxidant defences (e.g., HO-1, GPx, and SOD) and promotes autophagy via p62/SQSTM1. Together, the autophagy and antioxidant defences promote stem cell resistance to oxidative stress and limit cell death. This coordinated response preserves redox homeostasis and counteracts premature stem cell ageing. FOXO3 and Nrf2 thus represent promising therapeutic targets in age-related and oxidative stress-driven pathologies.

Transcription factors such as FOXO3 and Nrf2 play pivotal roles in mediating the crosstalk between autophagy and oxidative stress. FOXO3 is activated under oxidative stress and induces the expression of genes implicated in both autophagy and antioxidant defence (Figure 3). This includes upregulation of LC3 and BNIP3, which are critical for autophagy initiation and selective mitochondrial clearance [148]. Recent studies suggest that FOXO3 also regulates the transcription of enzymes like CAT and SOD, directly enhancing antioxidant capacity to mitigate ROS-induced damage. Gomez-Puerto et al. demonstrate that FOXO3 activation drives the expression of genes involved in autophagy, like MAP1LC3B, GABARAPL1, and PARK2, in hMSCs, and Zhang et al. reveal that FOXO3 induces autophagy through a transcription-dependent mechanism, requiring FOXO1 [53,149]. Overexpression of FOXO3 leads to increased transcription of FOXO1 and promotes its nuclear-to-cytoplasmic translocation, resulting in enhanced autophagy. The findings suggest a coordinated role of FOXO3 and FOXO1 in regulating autophagy in response to oxidative stress [149]. These studies collectively elucidate the mechanisms by which FOXO3 activation under oxidative stress conditions promotes Atg gene expression, ensuring cellular adaptation and stress resistance. Altogether, they highlight that FOXO3 reduces ROS levels by activating autophagy, underscoring its role in maintaining redox balance under oxidative stress conditions.

Similarly, Nrf2 coordinates the cellular response to oxidative stress by activating antioxidant response elements (AREs) in the promoters of genes coding for antioxidant enzymes, such as GPx and HO-1, as well as ATG proteins like p62/SQSTM1. Recent studies have elucidated the mechanisms by which, during oxidative stress stimuli, Nrf2 detaches from its inhibitor Keap1 and translocates to the cell nucleus to initiate transcriptional programmes that restore redox balance and promote autophagic degradation of damaged organelles [150].

In stem cells, the activation of these transcriptional programmes ensures adaptation to oxidative stress and maintenance of cellular homeostasis. FOXO3 activation has been shown to preserve HSC quiescence, while Nrf2 activation enhances MSC survival under oxidative conditions. For instance, FOXO3 plays a crucial role in preserving HSC quiescence under oxidative stress, acting as a prominent factor in the DNA damage response pathways of primitive hematopoietic cells, specifically in base excision repair, thereby protecting hematopoietic stem and progenitor cells from oxidative DNA damage during homeostasis [151]. Additionally, FOXO proteins, including FOXO3, exert critical roles in responding to physiological oxidative stress, not only mediating quiescence but also enhancing survival in the HSC niche. This function is crucial for maintaining the long-term regenerative potential of HSCs [152]. Furthermore, FOXO3 modulates ATM and oxidative stress, mediating distinct functions in regulating hematopoietic stem and progenitor cell fate [153]. These findings collectively underscore FOXO3′s pivotal role in maintaining HSC quiescence and protecting against oxidative stress, thereby ensuring the longevity and functionality of the hematopoietic system.

The differentiation of MSCs into osteoblasts necessitates a metabolic shift from glycolysis to enhanced mitochondrial respiration to meet the energy demands of this process. This metabolic transition leads to an increase in the production of endogenous ROS. Gomez-Puerto et al. investigated the role of FOXO3 in regulating ROS levels during osteogenic differentiation in hMSCs. Exposure to H_2_O_2_ triggered FOXO3 phosphorylation at Ser294 and its translocation to the nucleus, a process dependent on MAPK8/JNK activity. FOXO3 downregulation impaired osteoblastic differentiation and compromised the ability of hMSCs to regulate elevated ROS levels [53]. Additionally, they found that FOXO3 responds to increased ROS by inducing autophagy in hMSCs. Consistently, knockdown of ATG7 impaired autophagy, leading to disrupted ROS regulation and diminished osteoblast differentiation [53]. Collectively, these results support a model in which FOXO3 is essential for autophagy induction, thereby mitigating ROS accumulation resulting from heightened mitochondrial activity during osteoblast differentiation.

A few studies have investigated the role of Nrf2 activation in enhancing MSC survival under oxidative stress. One study demonstrated that transient overexpression of Nrf2 in MSCs safeguarded them from cell death activated by hypoxic and oxidative stress situations [154]. The activation of Nrf2 also augmented the activity of antioxidant enzymes like SOD and HO-1, contributing to increased cellular resilience [154]. Another recent research found that human umbilical cord-derived MSCs reduced high glucose-induced oxidative stress and prevented β-cell impairment through the activation of the Nrf2/HO-1 signalling axis [155]. A recent review emphasizes the crucial role of Nrf2 in maintaining MSC stemness, discussing how Nrf2 influences the self-renewal and differentiation of MSCs and contributes to their resilience under oxidative conditions [156]. These studies underscore the importance of Nrf2 activation in enhancing MSC survival under oxidative stress by upregulating antioxidant defences and maintaining cellular functions.

Emerging evidence highlights that these transcription factors do not act in isolation but form a tightly regulated network. Crosstalk between FOXO3 and Nrf2 allows for fine-tuning of autophagy and oxidative stress responses, ensuring optimal protection of stem cells from oxidative damage while maintaining their regenerative capacity [137]. This intricate regulatory interplay suggests that targeting FOXO3 and Nrf2 could be a promising therapeutic strategy to enhance stem cell resilience, particularly in the context of ageing and oxidative stress-related disorders.

### 4.3. Drugs Regulating Oxidative Stress and Autophagy Balance in Stem Cells

Several clinically used drugs modulate autophagy and oxidative stress, thereby interfering with key mechanisms that regulate stem cell viability and functions. Although originally developed for unrelated therapeutic purposes, many of these compounds exhibit pleiotropic effects on redox and mitochondrial homeostasis, ultimately impacting stem cell self-renewal, differentiation, and stress responsiveness either directly or indirectly.

Metformin, for instance, activates AMPK and inhibits mTOR phosphorylation, promoting autophagy and reducing mitochondrial ROS. In both in vitro and in vivo models, this has been associated with enhanced survival of NSCs and MSCs under oxidative stress conditions [157,158]. Statins likewise modulate redox signalling and have been implicated in the regulation of mitophagy and endoplasmic reticulum stress responses, particularly in vascular progenitor cells [159,160,161].

Interestingly, immunosuppressive drugs such as dexamethasone, widely used in clinical practice, in low dosage, have been shown to promote stem cell survival by inducing autophagy and fostering redox homeostasis [162,163].

Growing attention has been directed toward antioxidant compounds and natural polyphenols. Among natural compounds, resveratrol, a polyphenol well known in clinical treatments for its anti-inflammatory and immunomodulating properties, has been shown to modulate key cellular pathways involved in stem cell maintenance and function [164,165]. In mouse embryonic stem cells, resveratrol promotes pluripotency by activating AMPK/ULK1 and inhibiting mTORC1, thereby enhancing autophagy. This leads to upregulation of key pluripotency markers, improved mitochondrial function, and stabilization of the undifferentiated state under stress, supporting its potential as a tool to preserve stem cell identity in regenerative applications [166].

Melatonin, an endogenous hormone primarily secreted by the pineal gland, has emerged as a multifunctional modulator of autophagy and redox balance, playing a pivotal role in stem cell protection. In particular, it can exert a dual function: on one hand, it suppresses ROS production and activates antioxidant pathways such as Nrf2 and SOD2; on the other, it regulates autophagy in a context-dependent manner, thus promoting or suppressing autophagy by modulating the ERK/AKT/mTOR axis [167,168,169,170,171,172]. Indeed, in NSCs, melatonin promotes proliferation and differentiation under hypoxic conditions via ERK1/2 activation through the MT1 receptor [167], and counteracts toxin-induced excessive autophagy, such as that triggered by tri-ortho-cresyl phosphate, by restoring ERK1/2 signalling [173]. In pre-osteoblastic cells, it has been shown to suppress autophagy via ERK/AKT/mTOR pathway inhibition [171].

Similarly, melatonin exhibits potent antioxidant activity by reducing ROS production under both physiological and pathological conditions. In bone marrow-derived MSCs, it mitigates oxidative stress-induced apoptosis and enhances stem cell survival [172]. In NSCs, melatonin promotes proliferation and differentiation under hypoxia via ERK1/2 activation through the MT1 receptor [167]. Under inflammatory conditions, it upregulates SOX2 and HO-1 expression through PI3K/AKT-Nrf2 signalling, thereby supporting self-renewal while reducing apoptosis and aberrant differentiation [168,169,170].

Natural compounds such as icariin and curcumin have demonstrated protective effects in oxidative stress models by contextually modulating autophagy and redox responses. Icariin acts as a multitarget agent: it inhibits ROS-induced dysfunctional autophagy, enhances mitochondrial function, and activates PI3K/AKT/mTOR and MAPK/ERK pathways in EPCs and bone marrow MSCs [174,175], thereby reducing apoptosis and supporting cell survival, including in ischemic conditions [176].

Curcumin also exhibits a biphasic effect on autophagy. In NSCs, it reduces H_2_O_2_-induced ROS and malondialdehyde levels while inhibiting ERK1/2-mediated autophagy, thereby preserving cell viability [177,178]. Conversely, in adipose-derived stem cells, its protective effect is linked to autophagy induction and is abolished by 3-MA, indicating a cell-type- and microenvironment-dependent mechanism [179]. These findings highlight the importance of context-specific evaluation when considering curcumin for therapeutic applications.

### 4.4. Impact on Stem Cell Ageing and Regeneration

**Figure 4:**
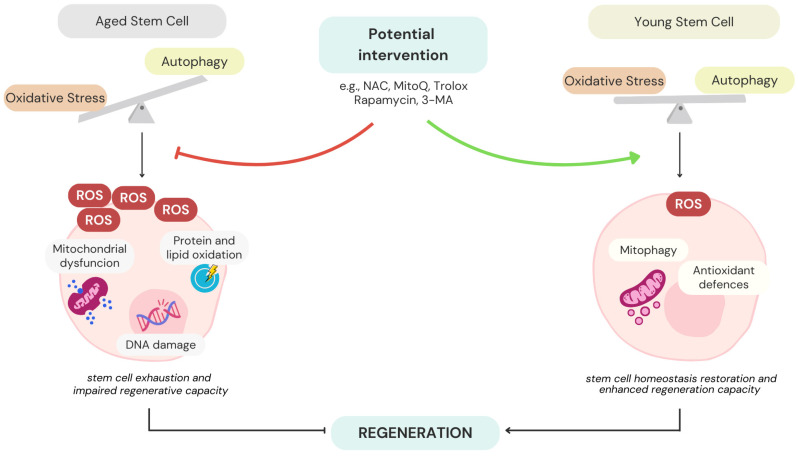
Pharmacological interventions targeting oxidative stress and autophagy to enhance tissue regenerative potential. Impaired autophagic activity and excessive oxidative stress in aged stem cells negatively impact their ability to regenerate. Elevated levels of ROS lead to mitochondrial dysfunction, as well as the oxidation of proteins and lipids, and DNA damage, ultimately resulting in stem cell senescence. In contrast, lower ROS levels combined with sufficient autophagic activity promote mitophagy and bolster antioxidant defences, which support stem cell renewal and differentiation, thereby enhancing tissue regeneration. Therapeutic agents such as NAC, MitoQ, Trolox, rapamycin, and 3-MA can help reduce oxidative stress and promote autophagy, ultimately restoring the homeostasis and regenerative capacity of stem cells.

The balance between autophagy and oxidative stress is particularly critical in the context of ageing. A decline in autophagic activity with age causes the build-up of damaged mitochondria and increased ROS levels, contributing to stem cell exhaustion and impaired regenerative capacity. Recent studies emphasized that the disruption of this balance accelerates cellular senescence and reduces the therapeutic potential of stem cells. Pharmacological interventions that restore autophagy or reduce oxidative stress have emerged as promising strategies to combat stem cell ageing (Figure 4).

For instance, activation of autophagy through compounds like rapamycin or resveratrol has been shown to rejuvenate aged HSCs through the improvement of mitochondrial functionality and reduction in ROS levels. These interventions not only restore self-renewal and differentiation potential but also enhance the overall longevity of stem cells [180,181,182]. HSC function declines with age, likely contributing to the weakened adaptive immune response seen in older individuals. Chen et al. discovered that mTOR activity is high in HSCs from aged mice compared to those from younger counterparts [180]. Inducing mTOR activation by conditionally deleting Tsc1 in young mice resulted in HSC characteristics resembling those of aged mice. These changes included increased expression of messenger RNA for the CDK inhibitors p16Ink4a, p19Arf, and p21Cip1, a decline in lymphopoiesis, and a reduced ability to regenerate the hematopoietic system [180]. In aged mice, treatment with rapamycin extended lifespan, restored HSC self-renewal and hematopoiesis, and improved vaccine response against a lethal influenza virus challenge [180]. These findings suggest that targeting mTOR signalling might be a promising therapeutic approach for counteracting age-related declines in HSC function. Further, it has been demonstrated that a three-week treatment with resveratrol increases both the frequency and total number of normal bone marrow HSC without affecting their competitive repopulation ability [181]. Furthermore, findings revealed that resveratrol enhances the multipotent progenitor capacity of bone marrow in vivo [181]. These results hold therapeutic potential for treating disorders related to HSCs and HSPCs, and may be valuable in bone marrow transplantation settings.

Similarly, mitochondrial-targeted antioxidants such as MitoQ and NAC have demonstrated efficacy in reducing oxidative damage and improving the survival of stem cells under stress conditions. These agents work by neutralizing ROS and supporting mitochondrial function, thereby maintaining the functional capacity of aged stem cells [183].

To investigate the role of ROS in vivo, Garcia-Prat et al. treated aged GFP-LC3 mice with intraperitoneal injections of the antioxidant factor Trolox, which enhanced autophagy and reduced senescence markers, such as p16Ink4a and senescence-associated β-galactosidase. Additionally, in vitro treatment with Trolox or p16Ink4a knockdown in MuSCs from aged and autophagy-deficient mice restored proliferation, delayed senescence, and improved regenerative potential [62]. More recently, in another in vivo study, the antioxidant icariin has been shown to promote the regeneration of pancreatic and germline stem cells, exert anti-inflammaging effects, and counteract age-related oxidative damage [184,185].

Additionally, the interplay between autophagy and oxidative stress has been related to the regulation of stem cell niche dynamics. Disrupted autophagy–oxidative stress balance can lead to an unfavourable niche environment, further impairing stem cell regeneration. Recent research underscores the role of Nrf2 and FOXO3 in modulating this balance to maintain a supportive microenvironment for stem cells. For example, Nrf2 activation has been shown to enhance the regenerative potential of mesenchymal stem cells in models of ageing and tissue repair [125].

The therapeutic implications of these findings extend to regenerative medicine, where targeting the autophagy–oxidative stress axis holds potential for improving the efficacy of stem cell-based therapies. Stem cell therapies have shown great potential as a therapeutic approach for several conditions associated with ischemic injury, such as ischemic stroke. However, a major challenge remains ensuring cell survival after transplantation. Recently, the effects of ischemia-induced oxidative stress were investigated by exposing human dental pulp stem cells (hDPSCs) and hMSCs to oxygen-glucose deprivation (OGD) [24]. This process led to excessive O_2_^•−^ and H_2_O_2_ production, which upregulated Ambra1 and Becn1 expression, thereby enhancing autophagy in both hDPSCs and hMSCs. Pre-conditioning with ROS scavengers significantly suppressed Ambra1 and Becn1 expression, confirming the role of O_2_^•−^ and H_2_O_2_ as upstream, unidirectional regulators of autophagy [24]. The involvement of ROS–p38–ERK1/2 signalling was further supported by the reversal of OGD-induced effects upon inhibition with SB202190 and PD98059, and by the observed activation of this pathway following SIRT3 depletion [24]. Global ROS inhibition with NAC, polyethylene glycol-SOD (PEG-SOD), or polyethylene glycol-CAT (PEG-CAT) further validated that elevated O_2_^•−^ and H_2_O_2_ impair stem cell viability via excessive autophagy. Notably, blocking autophagy with 3-MA markedly improved hDPSC survival [24]. These findings deepen our understanding of post-transplantation cell loss in hDPSCs and hMSCs and may inform new strategies to mitigate oxidative stress-induced therapeutic failure. 

By modulating critical pathways such as PINK1-Parkin-mediated mitophagy and Nrf2-driven antioxidant responses, it may be possible to extend the functional lifespan of stem cells and improve outcomes in ageing-related disorders and degenerative diseases [186]. These insights prepare for innovative therapeutic strategies aimed at restoring stem cell homeostasis and enhancing tissue regeneration in ageing individuals.

## 5. Conclusions and Future Directions

The insights gained from studying the interplay between autophagy and oxidative stress in stem cells have direct and promising implications for regenerative medicine.

Integrating targeted autophagy modulators with antioxidant-based strategies can enhance the effectiveness of stem cell therapies across a broad range of pathological conditions, including cardiovascular diseases, neurodegenerative disorders, and tissue injuries.

Despite substantial advances, critical aspects of the autophagy–oxidative stress crosstalk in stem cells remain incompletely understood. To fully exploit the therapeutic potential of redox–autophagy modulation in regenerative settings, several research directions should be prioritized. Among the most pressing challenges, a key priority is the need for in vivo studies employing conditional and lineage-specific deletion of autophagy-related genes (e.g., *Atg7* and *Atg5*) to clarify the intrinsic role of autophagy in the maintenance of adult stem cell compartments. Equally important is a deeper understanding of the tissue-specific dynamics of Nrf2 signalling and its interactions with other transcriptional regulators, which remain insufficiently characterized and currently limit the development of finely tuned redox-targeted therapies. Mitophagy also represents a critical yet underinvestigated target, with only a limited number of selective pharmacological modulators currently available, in contrast to the broader toolkit developed for macroautophagy.

A deeper understanding of how these pathways integrate within distinct stem cell niches and pathological contexts will be essential to minimize off-target effects and support the safe clinical translation of modulatory strategies. Given the tight interconnection of the signalling pathways involved, therapeutic manipulation of this axis may carry the risk of unintended disruptions to stem cell homeostasis. Advancing our mechanistic understanding of this complex network will be key to developing effective and safe regenerative interventions.

Upcoming research should focus on elucidating the context-specific mechanisms of this interplay and identifying novel therapeutic targets to harness these pathways for regenerative medicine in clinical settings.

## Abbreviations

The following abbreviations are used in this manuscript:

^•^OH	hydroxyl radicals
3-MA	3-methyladenine
AAP	ascorbic acid 2-phosphate
AKT	protein kinase B
ALA	α-lipoic acid
ALP	autophagy-lysosome pathway
Ambra1	activating molecule in BECN1-regulated autophagy protein 1
AMP	adenosine monophosphate
AMPK	AMP-activated protein kinase
AREs	antioxidant response elements
ASCs	adipose-derived stem cells
Atg	autophagy-related gene
ATG	autophagy-related protein
ATM	ataxia-telangiectasia mutated
ATP	adenosine triphosphate
Becn1	beclin-1
BMP4	bone morphogenetic protein 4
BNIP3	BCL2 Interacting Protein 3
BNIP3L	BCL2 Interacting Protein 3 like
CAT	catalase
CDK	cyclin-dependent Kinase
CDKIs	cyclin-dependent kinase inhibitors
c-MYC	cellular myelocytomatosis oncogene
CO_2_	carbon dioxide
CREB	cAMP response element-binding protein
DCF	dichlorodihydrofluorescein
DNA	deoxyribonucleic acid
DNMT1	DNA (cytosine-5)-methyltransferase
DPSCs	dental pulp stem cells
EPCs	endothelial precursor cells
EpiLCs	epiblast-like cells
ERK	extracellular signal-regulated kinase
ERRγ	estrogen-related receptor gamma
ESC	embryonic stem cell
FOXO3	forkhead box O3
FPN1	ferroportin 1
FUNDC1	FUN14 domain containing 1
GABARAPL1	GABA type A receptor-associated protein like 1
GATA4	GATA Binding Protein 4
GFP	green fluorescent protein
GPx	glutathione peroxidase
GSH	glutathione
GSK3β	glycogen synthase kinase-3 beta
H_2_O_2_	hydrogen peroxide
H3K4me3	tri-methylation of lysine 4 of the H3 histone protein
hDFSC	human dental follicle stem cells
hDPSC	human dental pulp stem cells
hESCs	human embryonic stem cells
HGF	hepatocyte growth factor
HIF1A	hypoxia inducible factor 1 subunit alpha
HK2	hexokinase
hMSCs	human mesenchimal stem cells
HO-1	heme oxygenase-1
HSCs	hematopoietic stem cells
HSP70	heat shock protein 70
HSPCs	hematopoietic stem and progenitor cells
hUCMSCs	human umbilical cord mesenchymal stem cells
IGF-1	insulin-like growth factor-1
iPSCs	induced pluripotent stem cells
JNK-1/2	c-Jun N-terminal kinases-1/2
Keap1	kelch-like ECH-associated protein 1
LC3	microtubule-associated protein 1A/1B-light chain 3
LDHA	lactate dehydrogenase
lncRNAs	long non-coding RNAs
LPS	lipopolysaccharide
LT-HSCs	long-term hematopoietic stem cells
MAP1LC3B	microtubule-associated proteins 1A/1B light chain 3B
MAPK	mitogen-activated protein kinase
mESCs	mouse embryonic stem cells
miRNAs	microRNAs
MitoQ	mitoquinone mesylate
MSCs	mesenchymal stem cells
MT1	melatonin receptor type 1A
mTOR	mechanistic target of rapamycin
mTORC1	mechanistic target of rapamycin complex 1
MuSCs	muscle stem cells
NAC	N-acetylcysteine
NADPH	nicotinamide adenine dinucleotide phosphate
NANOG	homeobox protein NANOG
NBR1	neighbour of BRCA1 gene 1
NDP52	pulp nuclear dot protein 52
NES	neuroepithelial stem
NF-κB	nuclear factor kappa B
NIX	nip3-like protein X
N-MYC	neuroblastoma-derived v-myc avian myelocytomatosis viral-related oncogene
NOS	nitric oxide synthase
Notch	notch receptor
NOXs	NADPH oxidase enzymes
NPCs	neural progenitor cells
Nrf2	nuclear factor erythroid 2-related factor 2
NSCs	neural stem cells
NSPC	neural stem and progenitor cells
O_2_	oxygen
O_2_^•−^	superoxide anions
OCT4	octamer-binding transcription factor 4
OGD	oxygen-glucose deprivation
*O*-GlcNAc	*O*-linked N-acetylglucosamine
Ogt	*O*-GlcNAc transferase gene
OGT	*O*-GlcNAc transferase
OxPhos	oxidative phosphorylation
PARK2	parkin-2 gene
PE	phosphatidylethanolamine
PEG-CAT	polyethylene glycol-CAT
PEG-SOD	polyethylene glycol-SOD
PGC-1α	peroxisome proliferator-activated receptor-gamma coactivator 1-alpha
piRNAs	piwi-interacting RNAs
PI3K	phosphatidylinositol 3-kinase
PIP3	phosphatidylinositol-3-phosphate
PINK1	PTEN-induced putative kinase 1
PKM1	pyruvate kinase M1
PKM2	pyruvate kinase M2
PRDX1/6	peroxiredoxins 1 and 6
PS1	presenilin 1
RNA	ribonucleic acid
ROS	reactive oxygen species
SASP	senescence-associated secretory phenotype
SCF	stem cell factor
SDF-1	stromal cell-derived factor-1
SIRT	sirtuin
SOD	superoxide dismutase
SOX2	sex-determining region Y-box 2
SQSTM1	sequestosome 1
Tat-Beclin1	mTORC1-independent autophagy activator
TCA-cycle	tricarboxylic acid cycle
TFEB	transcription factor EB
TOR	target of rapamycin
Tsc1	tuberous sclerosis gene
ULK1/2	unc-51-like kinase ½
VEGF	vascular endothelial growth factor
VPS15	phosphoinositide-3-kinase regulatory subunit 4
VPS34	phosphatidylinositol-3-kinase class III
WJ-MSCs	Wharton jelly multipotent stem cells
XO	xanthine oxidase
